# End-to-End Emulation of LoRaWAN Architecture and Infrastructure in Complex Smart City Scenarios Exploiting Containers

**DOI:** 10.3390/s24072024

**Published:** 2024-03-22

**Authors:** Massimiliano Gaffurini, Alessandra Flammini, Paolo Ferrari, Dhiego Fernandes Carvalho, Eduardo Paciencia Godoy, Emiliano Sisinni

**Affiliations:** 1 Department of Information Engineering, University of Brescia, 25123 Brescia, Italy; alessandra.flammini@unibs.it (A.F.); emiliano.sisinni@unibs.it (E.S.); 2 Department of Control and Automation Engineering, Sao Paulo State University, São Paulo 01049-010, Brazil; dhiego.fernandes@unesp.br (D.F.C.); eduardo.godoy@unesp.br (E.P.G.)

**Keywords:** smart city, LPWAN, distributed measurement system, virtualization, simulation, IoT

## Abstract

In a LoRaWAN network, the backend is generally distributed as Software as a Service (SaaS) based on container technology, and recently, a containerized version of the LoRaWAN node stack is also available. Exploiting the disaggregation of LoRaWAN components, this paper focuses on the emulation of complex end-to-end architecture and infrastructures for smart city scenarios, leveraging on lightweight virtualization technology. The fundamental metrics to gain insights and evaluate the scaling complexity of the emulated scenario are defined. Then, the methodology is applied to use cases taken from a real LoRaWAN application in a smart city with hundreds of nodes. As a result, the proposed approach based on containers allows for the following: (i) deployments of functionalities on diverse distributed hosts; (ii) the use of the very same SW running on real nodes; (iii) the simple configuration and management of the emulation process; (iv) affordable costs. Both premise and cloud servers are considered as emulation platforms to evaluate the resource request and emulation cost of the proposed approach. For instance, emulating one hour of an entire LoRaWAN network with hundreds of nodes requires very affordable hardware that, if realized with a cloud-based computing platform, may cost less than USD 1.

## 1. Introduction

Modern distributed IoT applications rely on the pervasive deployment of sensors, computing resources, and microservices. The free combination of these three parts enables innovative services. Computing can be available in both cloud servers (cloud computing) and in other devices physically close to sensors (edge computing). Cloud computing offers unlimited computational power for scaling high-latency services, while edge technology is used for low-latency services [1]. In parallel, the need for the extreme flexibility and fluidity of IoT applications pushes the development platform for easing the management and the update of distributed microservices/functions. In the last few years, containerization technology has been used as a lightweight virtualization (LV) method, guaranteeing the portability of processes and microservices between different computational platforms [2]. For instance, in a growing IoT scenario, containers can be seamlessly moved from the cloud to the edge and from one hardware device to another.

The IoT paradigm also poses constraints on communication technology. The LoRaWAN belongs to the LPWAN (Low-Power Wide-Area Network) family [3]. It has been designed to provide long-range wireless connectivity to simple IoT nodes, and for this reason, its complexity is asymmetrically distributed: the protocol stack in the node is simple, while most of the network functions are implemented in the backend servers. In other words, in the original LoRaWAN concept, computational power is only in the “backend”. LoRaWAN systems, in the past, took advantage of these new trends, and for instance, the LoRaWAN backend services are available as containers for deployment on a cloud/premise server. On the other hand, LoRaWAN nodes are still provided as monolithic hardware modules or as stack libraries for low-level embedded systems with specific transceivers.

Recently, the authors proposed a containerized version of the LoRaWAN end node functionalities. All the node stack functions are now available as containerized microservices that can be deployed in any edge device (even in devices already present in the destination environment). Hence, the main advantage is that the dependency on a specific hardware and, even, the dependency from a specific wireless technology are overcome. Moreover, thanks to the containerization of both nodes and the backend, the complete disaggregation and decomposition of the LoRaWAN architecture is now available. With these basic “bricks”, innovative end-to-end full-stack containerized LoRaWAN systems can be implemented.

This paper is focused on the emulation of such kinds of innovative systems. As a matter of fact, since each part of the system is containerized, the emulator can run each service as it was running on the target device, enabling a very realistic emulation. In this case, extremely valuable insights for optimizing resource utilization and performance in a smart cities scenario can be obtained even before deploying any real hardware devices.

Clearly, the overhead of the virtualization may burden in excess the simulation host, and new methodologies to estimate the emulation complexity must be researched.

As matter of fact, the use of LV and containers and, more in general, the split of stack functionalities into separated microservices has already been suggested for implementing emulation platforms [4], since it favors the decoupling of the “software” components from the underlying “hardware” equipment.

However, there have been no attempts in the literature addressing a complete, end-to-end emulation of LoRaWAN networks that exploit disaggregation and decomposition at both the field and the backend sides. This work tries to fill this gap, paying attention to the actual scalability of the emulated network in a relatively simple but meaningful scenario.

For these reasons, the goals of this paper are as follows:

- The highlighting of the differences between traditional network simulation and the emulation approach required to gain more insights on innovative end-to-end full-stack containerized systems.
- The characterization of simulation systems that can emulate an entire end-to-end LoRaWAN architecture built with the innovative end-to-end full-stack containerization.
- The definition of the fundamental evaluation metrics for comparing different implementations.
- The evaluation, and the comparison, of five use cases inspired by a smart city scenario in order to highlight the effectiveness of the proposed approach.

This paper is arranged as follows. In Section 2, related works are briefly discussed. In Section 3, lightweight virtualization and containers for emulation are introduced. In Section 4, the reference smart city scenario is briefly described. In Section 5, details about the LoRaWAN and container-based disaggregation are provided. In Section 6, the emulation of entire LoRaWAN networks is presented, while in Section 7, the results for the proposed use cases are resumed and discussed. Finally, conclusions are drawn.

## 2. Related Works

This section provides an overview of the literature about existing solutions and frameworks for evaluating the performance of networked systems. In particular, depending on how nodes are actually modeled, it is possible to group the diverse solutions into four main categories: (i) (system-level) simulators; (ii) emulators; (iii) hybrid approaches; and (iv) prototype-based demonstrators. As regards simulators, a high-level behavioral description of the node is used, disregarding fine-grained details. In emulators, the node model is closer to the real-world implementation, possibly sharing some sections of the code; the main difference is in the hardware used and the impact of the environment. Hybrid approaches mix the emulated and simulated nodes in a single framework. Finally, a proof-of-concept can be realized to test performance in the real world.

System-level simulators (SLSs) enable system designers to gauge the effects of variables such as node density or traffic loads, uplink and downlink traffic interaction, macro-diversity, and medium access protocols on system performance. The most widely used simulation engines are OMNeT++ [5] and ns3 [6], both using C++ for model implementation. Recently, solutions based on the SimPy framework have been proposed, leveraging Python [7].

Regarding emulators, the idea of reusing the very same code of the real nodes for evaluation purposes is not new at all [8]. Generally speaking, so-called lightweight virtualization (LV) is used for isolating virtual nodes, allowing them to coexist in the same host. Mininet/Mininet-WiFi [9,10] and IMUNES [11] are well-known examples of such an approach, targeting IP-based communication protocols. In particular, the latest Linux-based IMUNES release generalizes LV exploiting Docker-based containers. Interesting to note, when WiFi wireless networks are of interest, a simplified channel model is natively provided by the Linux kernel via the hwsim0 virtual interface [12]. The need for realistic and reproducible “virtual” experiments for evaluating network behavior in terms of functionalities, timings, and exchanged traffic is stressed in [13], where the adoption of the container-based Mininet-HiFi emulator is suggested due to the resource isolation, provisioning, and monitoring mechanisms it offers.

Authors of [14] proposed Dockersim, which merges advantages of Docker-based LV and the OMNeT++ simulator. A similar approach is implemented in Dockemu [15], merging Docker-based LV and ns3; in this case, Tap net devices offered by the simulator, which permits the integration of real-world internet hosts (supporting Tun/Tap devices) into the simulator, are used as well. Indeed, including virtual and physical nodes in emulation frameworks is particularly important when features such as dependability and scalability are of interest [16]. As a matter of fact, the use of LV and containers can facilitate this integration and enable the implementation of hybrid simulation/emulation platforms that exploit the microservices paradigm, e.g., as shown in [17].

It is useful to highlight how the need for evaluating very large testbeds, as dictated by IoT-like applications [18], required the revision of framework implementation exploiting the latest development in LV, as for Katharà [19]. As a consequence, identifying bottlenecks and evaluating performance offered by these platforms assumed higher and higher relevance. Several research efforts have been carried out in this direction, e.g., as reported in [20].

As a resuming remark, it must be stressed that the success and widespread adoption of LV and containerization for implementing emulators must be traced back to the use of the very same technologies in setting up complex and heterogeneous applications operating at the cloud, fog, and edge levels [21], as occurs for 5G deployments [22].

Focusing on LoRaWAN, it is worth mentioning FLoRa [23], based on the OMNeT++ engine and the INET framework; LoRaSim [24], a discrete event simulator exploiting the aforementioned SimPy Python engine; and the LoRaWAN modules implemented in ns3 [25]. A more exhaustive overview of simulation and emulation platforms targeting LoRaWAN is described in [26]. It must be stressed that IoT-like applications often involve hundreds, if not thousands, of end devices, and the overall performance can be severely affected by the scalability offered by the LoRaWAN technology. For instance, in [27], the analysis of the scalability in large-scale LoRaWAN networks is addressed using the ELoRa framework, based on the aforementioned ns3 simulator. However, as previously stated, despite simulation models being able to faithfully mimic the ideal behavior of a LoRaWAN device, they could completely neglect the non-idealities and bottlenecks of the actual real-world protocol stack implementations.

Nowadays, a complete emulation platform targeting LoRaWAN is missing, at least according to the authors’ best knowledge, but attempts have been discussed in the past to merge real LoRaWAN backend with purposely designed traffic generators able to mimic the huge number of messages generated by such dense networks. An example is the previously mentioned ELoRa; interesting to note, open-source solutions exist as well, like the LoRaHammer (available online at: http://lorhammer.itk.fr/ (accessed on 4 March 2024)) and the LWN (available online at: https://github.com/UniCT-ARSLab/LWN-Simulator (accessed on 4 March 2024)). Additionally, the Mbed Simulator (available online at: https://os.mbed.com/blog/entry/introducing-mbed-simulator (accessed on 4 March 2024)) permits the execution of code developed for the Mbed-OS from ARM, claiming to be an open-source, easy-to-use operating system purposely designed for the IoT and natively supporting LoRaWAN, in a confined simulated environment.

The authors aim to fill this gap by mimicking the approach in Mininet/Mininet-WiFi and IMUNES frameworks in the LoRaWAN case. In particular, the disaggregation and decomposition of end devices, enabled by container-based LV, are proposed for end devices, which can be easily integrated with backends that natively exploit the microservices paradigm, in a complete emulation solution.

As a final remark, it must be noticed that the performance indicator for emulation systems can be very complex, as in Rak [28], requiring deep knowledge of the application level, a situation that does not fit the current research of this paper. However, knowledge about the number of manageable emulated end devices would be of main importance. Nevertheless, there is no evidence in the literature about the resource requirements or the cost of such a solution when deployed in the cloud. The authors also address this problem, demonstrating the feasibility of emulating a dense LoRaWAN network in a typical scenario.

## 3. Lightweight Virtualization and Containers as Emulation Enablers

**Figure 1:**
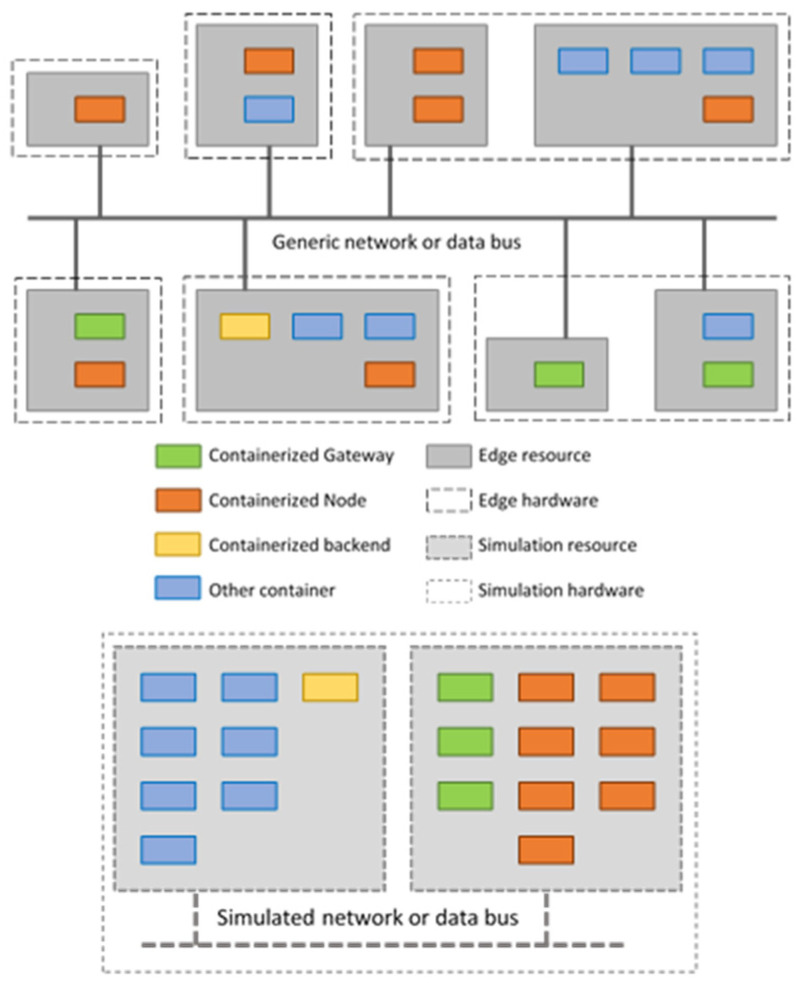
An overview of the considered emulation scenario (**bottom**), sharing the same container images of a real edge deployment of the proposed full-stack containerized LoRaWAN (**top**).

As stated in the Introduction, this work proposes to accurately emulate heterogeneous systems exploiting communication technologies using the very same containerized services they are based on. As shown in Figure 1 (top), a very complex arrangement can be devised, where multiple edge-class physical machines host several edge resources, e.g., regular virtual machines abstracting the underlying hardware. In turn, considering microservice architecture, each of these edge resources host several LV-containerized applications.

However, it has to be highlighted that the aim of disaggregating and decomposing network functionalities is to split the software components, which actually describes the functionality of interest, from the underlying hardware equipment. According to the microservices paradigm, it means that a single container can be devoted to each disaggregated and decomposed functionality. This separation is of paramount importance for system emulation, suggesting that the same method can also be used for developing an emulation solution, as shown in Figure 1 (bottom). The interesting thing to note is that the emulated scenario shares the same container images of the real-world deployment.

Generally speaking, the goal of all emulation systems is to execute experiments in a realistic and reproducible network setup. More in detail, the realistic behavior of the emulated architecture can be expressed in terms of the following:

- Functionality (i.e., replicate the same functionality of real hardware in a real deployment, executing the same code);
- Timing (i.e., timings must be as close as possible to the deployed hardware);
- Generated traffic (i.e., real traffic must be managed, possibly including traffic to and from external sources).

In addition, focusing on communication architecture, the features of interest can be expressed in terms of the following:

- Topology flexibility (i.e., different topology should be supported);
- Reproducibility (i.e., it should be simple to replicate experiments);
- Cost (that should be as low as possible).

Clearly, there are also shortcomings associated with the container-based approach, such as the following:

- The level of isolation among containers, which could interfere with each other;
- Differences between the OS of the containers are less evident;
- The deployment of the testbed could take a long time and, if automated, could hide inefficiencies;
- Timing fidelity could be compromised. Resource exhaustion in the host can reduce throughput and cause non-real-time situations; while in the opposite case (i.e., when the emulation platform has oversized computational and storage resources), shorter task executions with respect to real hardware components could happen;
- The emulated channel approximates the real channel (especially for wireless communications).

### The Proposed End-to-End LoRaWAN Emulation

When LoRaWAN technology is of interest, the containers allow for the implementation of logical entities we find in the backend, i.e., the so-called Network and Application Servers, connected by a generic data bus to allow for information exchange. The same approach is also currently used for implementing gateways. In light of this assessment, the authors already also extended this approach to LoRaWAN end nodes [29].

In this work, these LV containers are exploited to develop a complete LoRaWAN emulation solution, including both the infrastructure (i.e., the backend servers and the gateways) and the end devices.

The mapping of the indicators introduced above can also be applied to the LoRaWAN use case considered in this paper, as reported in Section 6.

In particular, the price to pay for enhanced flexibility is, among other things, the larger resource requirement. However, constraints dictated by the containerization overhead are not reported in the literature, at least in a dense scenario and particularly for LoRaWAN. For this reason, a set of easy-to-compute metrics has been identified and meaningful, but general, use cases have been designed to quantify the scalability of such an arrangement.

## 4. An Example of a Smart City Scenario: The A2A Smart City

The smart city scenario is nowadays embraced by most multi-utility companies all around the world, seeking efficiency improvement and waste minimization.

A2A is currently the largest multi-utility group in Italy; it is involved in generating and distributing electricity, also from renewable sources, gas and in integrated water supply; it is a leader in Italy for district heating, and waste management services, where it applies the circular economy approach for improving the overall efficiency and reducing the impact on the environment. The company has significant presence in the north of Italy and can boast its own power plants facilities in Italy and Greece.

The A2A Smart City initiative launched in 2016, finally merged into the A2A Smart City subsidiary located in Brescia, in order to promote the adoption of the IoT paradigm in the smart city application scenario. Since the very beginning, LoRaWAN was chosen for the communication infrastructure and a competence center, named “Smartcity LAB”, was created to test the suitability of LoRaWAN for different use cases and smart environments, like smart parking, smart metering, smart bin, and so on [30].

The company also proposes its own LoRaWAN backend, after the acquisition, in 2017, of the Patavina Technology spin-off from the University of Padua. The backend is offered to users according to the Platform as a Service (PaaS) scheme. The backend consists of the PTNetSuite network manager (i.e., the Network Server defined in Section 5.1), which allows for the integration of private GWs or the exploitation of public ones. The PTNetSuite is interfaced with the CityEye platform (i.e., the Application Server defined in Section 5.1), for sensor data preprocessing and user-friendly, interactive dashboards showing sensors’ locations and output values. Additionally, REST-APIs are available for retrieving data, enabling further analyses and/or premises storage. Currently, A2A Smart City has about 3000 installed sensors and hosts a publicly accessible AS including more than 500 devices, which can be reached at the Cityeye frontend website (available online at: https://www.cityeye.it (accessed on 4 March 2024)).

As a resuming remark, it can be affirmed that the A2A Smart City solution previously described further confirms that real-world applications in the context of smart grids may involve a relevant number of end devices, which could be hard to be managed by using an emulation solution. For this reason, in this paper, we investigate the actual scalability of the LV-based end-to-end emulation of LoRaWAN, filling a gap in the available literature.

## 5. Communication Infrastructures: The LoRaWAN Case

This section aims at furnishing a brief overview of the considered technologies, LoRaWAN and LV, and their use for disaggregating and decomposing the devices and the backend.

### 5.1. The LoRaWAN Communication Solution

LoRaWAN networks implement the star-of-stars topology, in which gateways (GWs) are all connected to the same backend and also represent the center of the (wireless) stars. The wireless up- and downlink leverage proprietary LoRa modulation, an enhanced Chirp Spread Spectrum modulation that allows for the coding of the SF (the Spreading Factor) bits per symbol in the channel bandwidth B_CH_. Depending on the SF ∈ {7, …, 12} and on the B_CH_ ∈ {125, 250} kHz, the actual data rate can range from about 11 kbps (SF = 7 and B_CH_ = 250 kHz) down to about 0.3 kbps (SF12 and B_CH_ = 125 kHz).

**Figure 2:**
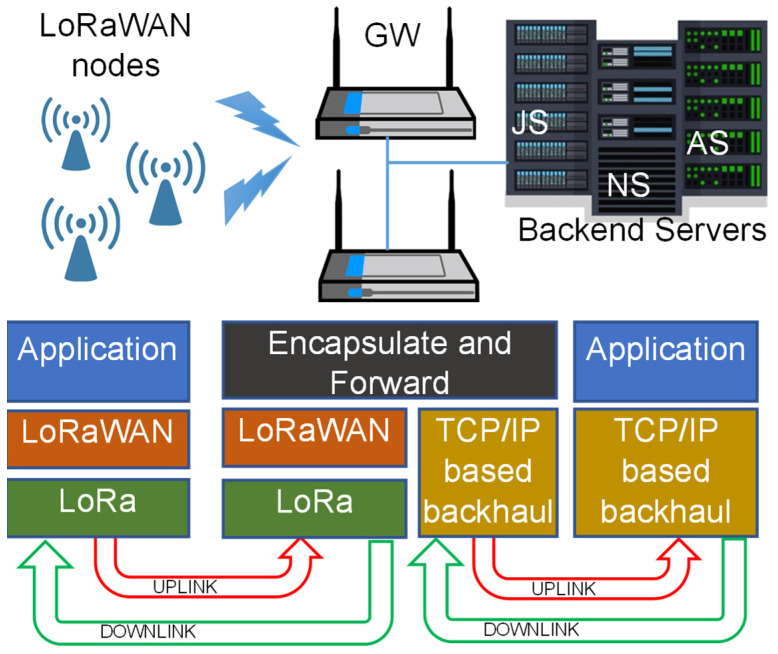
The LoRaWAN architecture.

IP-based (wired) networks are generally adopted for connecting the GWs and the backend. The latter hosts the following: (i) the Network Server (NS), for network management, including adaptive data rate strategies; (ii) the Application Server (AS), for application data management, including integration with end-user applications; and (iii) the Join Server (JS), for managing security and encryption keys (indeed, the JS has been introduced only in recent LoRaWAN releases). A pictorial description of the LoRaWAN network architecture is depicted in Figure 2.

### 5.2. Lightweight Virtualization

Virtualization is the process of abstracting underlying hardware into a virtual machine instance, allowing for a set of processes to believe they are executed by a dedicated system. Heavyweight approaches involve a separate operating system (OS) kernel for each virtual machine, ensuring the strong segregation of execution environments but demanding high computational and storage resources. On the other hand, lightweight virtualization (LV) is typically based on container concepts, wherein processes within a container share the same kernel, resulting in improved performance. LV maintains virtualized instance isolation but offers faster creation and initialization. This is particularly advantageous in Internet of Things (IoT) scenarios, where numerous applications can be virtualized on the same host due to small container image footprints and minimal resource requirements [31].

Containerization leverages features of the operating system kernel, such as namespaces and control groups (cgroups). Linux namespaces ensure container isolation, while cgroups allow for resource reduction per container. Containers can be application- or system-oriented, depending on whether they execute a single or multiple applications. In this context, application-oriented Docker containers are employed to map LoRaWAN node functionalities into a set of microservices. Docker containers are managed by the underlying container engine using a client–server approach through APIs and a command-line interface.

Containerized applications typically communicate through (virtual) IP-based networking, facilitating easy connection between them and interaction with other workloads, including external ones. Docker’s networking subsystem, based on drivers, provides varying levels of isolation from the host network. Above this virtual network, message-oriented middleware can be utilized, mirroring the strategy employed for IoT device interaction. A common example is the use of MQTT, which requires an additional containerized broker.

### 5.3. LoRaWAN Devices’ Disaggregation and Decomposition

In the previous subsection, we underscored the advantages offered by LV, potentially utilizing containers. For similar reasons, most, if not all, contemporary LoRaWAN backends typically adopt a modular architecture, wherein individual blocks are implemented within a single container, aligning with the microservices approach. Prominent examples include The Thing Stack (https://www.thethingsindustries.com/docs/the-things-stack/host/docker/, accessed on 4 March 2024), the open-source community offering The Things Industries enterprise solution (distributed under the Apache 2.0 license), and ChirpStack (https://www.chirpstack.io/docs/getting-started/docker.html, accessed on 4 March 2024), another open-source solution (distributed under the MIT license).

**Figure 3:**
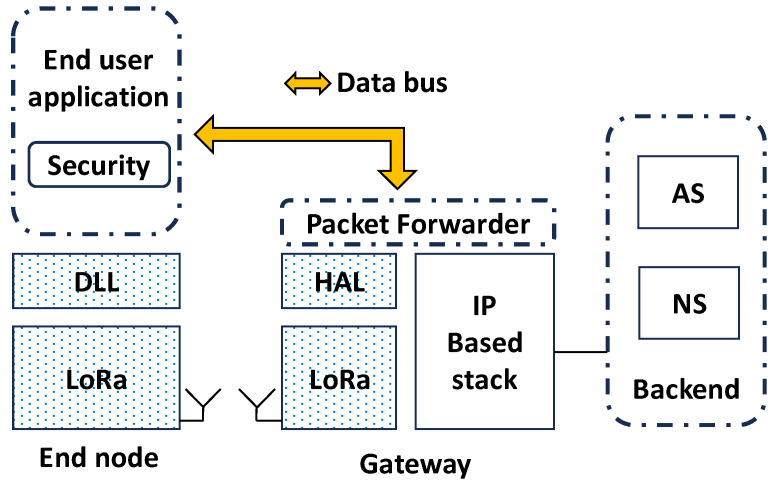
The fully virtualized scenario: rounded, dotted boxes represent containers. Grayed-out boxes represent the LoRaWAN and LoRa functionalities no longer needed (DLL: Data Link Layer; HAL: Hardware Abstraction Layer).

In this work, LV is addressed for the disaggregation and decomposition of end device functionalities using containers [29]. In particular, a fully virtualized scenario can be devised, as shown in Figure 3, where LoRaWAN compliant messages are exchanged between virtualized end node(s) and GW(s) across the data bus provided by the chosen LV framework.

**Figure 4:**
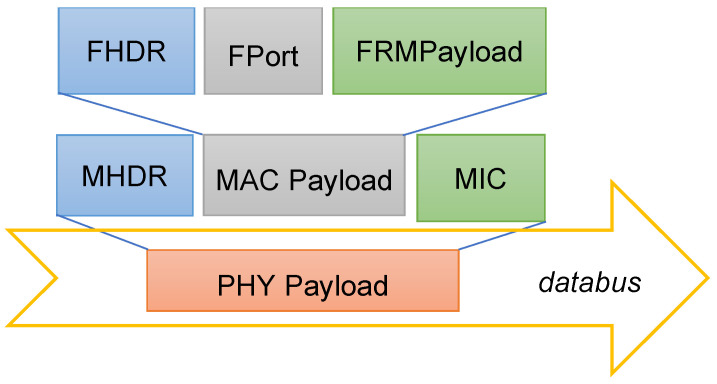
LoRaWAN message fields (PHY header and trailer depend on LoRa and are not shown since they do not apply to “Full” virtualized scenario).

Therefore, the LoRaWAN physical layer payload (PHYPayload in Figure 4) is generated by the (single) container providing end node services and exchanged with another container responsible for packet forwarder functionalities, through the shared data bus.

It must be noted that the use of de facto standard packet forwarder protocols, like the Semtech UDP one, ensures compatibility with regular backends. In particular, as previously mentioned, most, if not all, open-source backends can be easily integrated into the proposed emulation solution. A positive side effect is that compliance with the LoRaWAN standard is therefore ensured.

The PHYPayload includes three fields: the MAC Header (MHDR), providing the message type and additional metadata; the actual message (MACPayload); and the Message Integrity Check (MIC). The MACPayload includes three significant fields: the Frame Header (FHDR); the Port field (Fport), containing the device address, a frame control field, a frame counter field, and an options field; and the MAC Frame Payload (FRMPayload). The Fport identifies MAC commands (for the NS) and user data (for the AS). For security reasons, the FRMPayload is ciphered, and the MIC is computed to allow for an integrity check at the NS. The relevant session keys are retrieved thanks to the LoRaWAN provisioning mechanism, which is in most cases the Over-The-Air Activation (OTAA).

All the provisioning and the security-related tasks are managed by the containerized virtual node, mimicking the behavior of a real node. On the contrary, the message content is opaque for the packet forwarder, which is only in charge of dispatching the messages to the desired NS. Notably, in this configuration, the physical layer, i.e., the LoRa radio, is not considered at all. In turn, it means that the timing constraints dictated by LoRa communications do not apply to this scenario.

The Full approach is particularly compelling due to its remarkable flexibility. It establishes a virtualized LoRaWAN network that retains the use of a standard backend while accommodating any data bus, such as one based on message-oriented middleware over a conventional TCP/IP network. An additional advantage of the considered approach is that containers do not need to be instantiated on the same hardware but can be executed on different computational nodes. For instance, the end-user application and security management containers could run on one device, while the packet forwarder container operates on another device.

Applications that can profit from these advantages include prototypal systems for development and debugging purposes and heterogeneous systems in which different communication solutions must coexist (e.g., for backup or retrofitting) [32].

## 6. Emulation of End-to-End LoRaWAN Infrastructure

The goal of this section is to define the figure of merits evaluated during the LoRaWAN network emulation and the definition of meaningful use cases.

### 6.1. Performance-Related Metrics

In order to quantitatively evaluate the impact of container-based LV used for nodes and backend implementations, several metrics have been considered. In particular, the overhead can be estimated by comparing the offered load for the different scenarios in terms of a statistical analysis of the following:

- **CPU**: CPU usage, which is the percentage (%) of the overall host per CPU capacity used by a single container, i.e., the percentage of the host per CPU time being used by the container;
- **MEM**: memory usage, which is the overall host memory capacity used by a single container expressed in megabytes (MB);
- **NET**: network usage, which is the amount of input/output traffic exchanged across the network interface of a single container expressed in kilobytes (kB).

Data written to or read from the host by containers are not relevant to the proposed implementation, as it does not involve the use of volumes.

However, it would be interesting if the metrics could be evaluated easily and without affecting/modifying the underlying LV engine. Fortunately, Docker natively offers the docker stats utility that provides a periodically updated set of average metrics’ values per container being executed. Therefore, the command can be repeatedly issued to (asynchronously) sample the metrics’ average and collect a statistically significant data set.

Besides the previous container-oriented metrics, the overall performance of the entire emulation is expressed using the following metrics:

- **TCPU**: the total CPU usage of the overall host machine during the emulation per CPU capacity, i.e., the percentage of the host per CPU time being used by all the emulation processes;
- **TMEM**: the total memory usage, which is the percentage of the overall host memory capacity used during the emulation expressed in megabytes (MB);
- **TNET**: the total network usage, which is the amount of input/output traffic exchanged across the network interface during the emulation expressed in kilobytes (kB).

### 6.2. Utility “Docker Stats”

The docker stats (https://docs.docker.com/config/containers/runmetrics/ (accessed on 4 March 2024)) is a Docker command that returns a live data stream of diagnostic data for each running container. The returned values are as follows:

- CONTAINER ID and NAME, the ID and name of the container;
- CPU % and MEM %, the percentage of the host’s CPU and memory the container is using;
- MEM USAGE/LIMIT, the total memory the container is using, and the total amount of memory it is allowed to use;
- NET I/O, the amount of data the container has received and sent over its network interface;
- BLOCK I/O, the amount of data the container has written to and read from block devices on the host;
- PIDs, the number of processes or threads the container has created.

**Figure 5:**
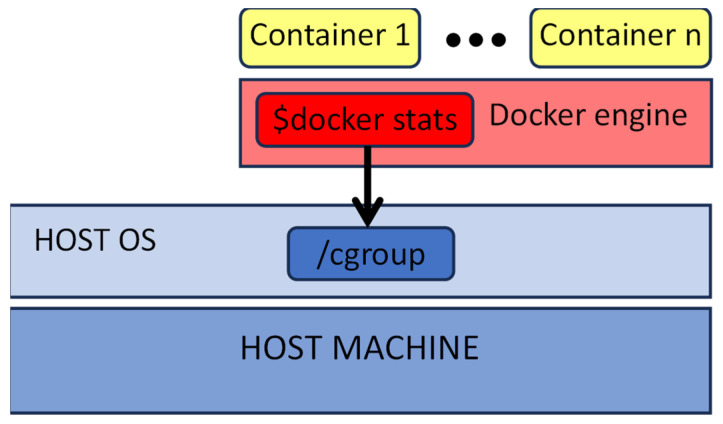
Monitoring infrastructure.

As shown in Figure 5, docker stats runs in the Docker engine and it queries directly /cgroup hierarchy, to analyze metrics such as CPU usage and MEMory usage, as reported for different cgroup versions and drivers:

- /sys/fs/cgroup/memory/docker/<longid>/ on cgroup v1, cgroupfs driver;
- /sys/fs/cgroup/memory/system.slice/docker-<longid>.scope/ on cgroup v1, systemd driver;
- /sys/fs/cgroup/docker/<longid>/ on cgroup v2, cgroupfs driver;
- /sys/fs/cgroup/system.slice/docker-<longid>.scope/ on cgroup v2, systemd driver.

In order to correctly understand how the stats are evaluated, the source code of the Docker CLI (https://github.com/docker/cli/blob/6c12a82f330675d4e2cfff4f8b89a353bcb1fecd/cli/command/container/stats_helpers.go#L180 (accessed on 4 March 2024)). In particular, Equations (1)–(4) report the relations used to estimate runtime statistics taken into account in Section 6.1:

“CPU %” = (cpuDelta/systemDelta) × onlineCPUs × 100.0(1)

where for each container cpuDelta is obtained by subtracting the previous total used CPUs’ time from the current total used CPUs’ time, and systemDelta is calculated by subtracting the previous total CPUs’ usage time to the total host CPUs’ usage time. Each value represents the delta usage expressed in nanoseconds. The onlineCPUs is the number of CPUs that the container is using; multiplying it by the ratio between the CPU occupied by the container and the total system CPU has the effect of moving the full scale.

“MEM USAGE” = memTotalUsage − memUsageCache(2)

where memTotalUsage is the total amount of memory that the container is using, and memUsageCache is the cache usage.

Differently from “CPU %” and “MEM USAGE”, network metrics are not exposed by cgroup. Network statistics are collected using the netstat command (https://linux.die.net/man/8/netstat, accessed on 4 March 2024) in the network namespace of the container:

NET_I += RxBytes(3)

NET_O += TxBytes(4)

RxBytes and TxBytes are, respectively, the amounts of received and transmitted bytes from the virtual Ethernet interface of the container.

To obtain the metrics described in the previous section, Equations (5)–(7) are used. The total network traffic, NET, results from summing the values obtained from Equations (3) and (4); the CPU indicator is normalized to the number of onlineCPUs, while the MEM indicator is directly derived from the memory usage:

NET = NET_I + NET_O(5)

CPU = “CPU %”/onlineCPUs(6)

MEM = “MEM USAGE”(7)

### 6.3. Definition of the Simulation Use Cases

**Figure 6:**
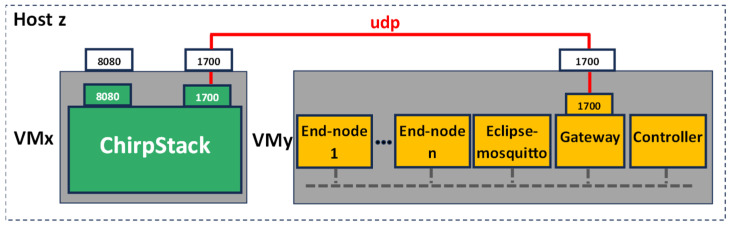
Proposed use case topological view.

Without losing generality, the open-source ChirpStack solution has been considered as the reference LoRaWAN backend. In Figure 6, the topological view of the proposed emulation setup is shown. There are two virtual machines, called ‘VMx’ and ‘VMy’, respectively, each one representing an edge-like computing resource and hosted in the same physical machine ‘HostZ’. In the VMx, it is virtualized by the LoRaWAN backend; despite it consisting of several containers, it has been represented by a single ‘ChirpStack’ block in Figure 6, for the sake of simplicity. The VMy hosts the fully virtualized scenario previously introduced in Section 5.3; in particular, several ‘End node’ and ‘Gateway’ containers are recognizable in Figure 6. The data bus leverages the MQTT protocol; accordingly, an ‘Eclipse-mosquitto’ container, image version 1.6.14, is also present for providing the broker functionalities. On the other hand, the connectivity between the backend (in the VMx) and the gateway (in the VMy) exploits the well-known Semtech UDP (https://github.com/Lora-net/packet_forwarder/blob/master/PROTOCOL.TXT (accessed on 4 March 2024)) protocol; for this reason, the port 1700 is exposed on both VMs, and a virtual network implemented by the hypervisor connects them.

The VMy features a last container, named ‘Controller’. The Controller is the manager of the emulation. It generates the process values (i.e., the high-level information to be transmitted) and activates the corresponding LoRaWAN end node, which is, in turn, the sole node responsible for creating the uplink message and forwarding it to the gateway. In the proposed emulation setup, without loss of generality, the Controller is implemented by means of Node-RED Flow programming. In detail, the Controller publishes a new specific MQTT topic each time the end node service must be triggered to send a new uplink message.

Since the aim of this paper is to assess the occupation of hardware resources of the host, no resource limitation or reservation for the services are set in the docker-compose file.

Metrics’ evaluation and analysis in this work are focused on containers hosted by the VMy, which are the components permitting the proposed innovative full-stack emulation.

For the sake of completeness, two different emulation platforms, both based on the topology previously described, have been addressed.

- The first scenario, called Local, corresponds to an on-premises platform. Two scenarios are investigated, as reported in Table 1: local use case 1 and local use case 2. The two use cases differ from the host hardware setup and the virtualization software to the virtual machines’ configuration. The aim of this scenario is to evaluate the impact of emulation on different hardware and software configurations.
- The second scenario, called Cloud, corresponds to a cloud server platform; in particular, without loss of generality, Google Cloud is considered. Three scenarios are investigated, as reported in Table 2: Cloud use case 1, cloud use case 2, and cloud use case 3. Different from the Local scenario, Cloud is not interested in investigating the difference between host configuration but is focused on evaluating the resource and cost utilization during the different emulations. The various cloud use cases differ from the cost per hour, the number of vCPUs, and the series. Please note that the reported costs may be tied to specific contracts with Google Cloud, so they are used here only for reference purposes.

### 6.4. Threats to the Validity of the End-to-End LoRaWAN Emulation

The very same considerations reported in the Section 3 discussion also apply to the LoRaWAN emulation use cases previously described, as shown in Table 3.

The analysis further confirms the strength points of the LV container-based emulation. In particular, it can be highlighted that moving the emulation in the cloud is expected to have better timing fidelity, due to the larger amount of available resources and lower (fixed) cost, due to the pay-per-use subscription. Since channel modeling is out of this paper’s scope, related requirements cannot be evaluated.

## 7. Use Case Results and Discussion

Inspired by the size and complexity of the smart city scenario described in Section 4, this section defines the different sets of parameters applied to the use cases of Section 6.3, and then it illustrates their results.

In all the use cases, a new LoRaWAN uplink message is triggered by the Controller every 20 s per each node. This means that the considered scenarios are symmetric from the data-per-node point of view. All the experiments reported in this work refer, without loss of generality, to the emulation of a full LoRaWAN architecture operating for 15 min with a variable number of end nodes N ∈ [1, 2, 4, 8, 16, 32, 64, 128, 256, 512].

Note that the configurations of all the components described in Section 5 are the default ones, in order to allow for the simple reproduction of the experiments. Hence, better numerical results could be obtained by the fine-tuning of parameters, but this is out of the scope of this paper.

**Figure 7:**
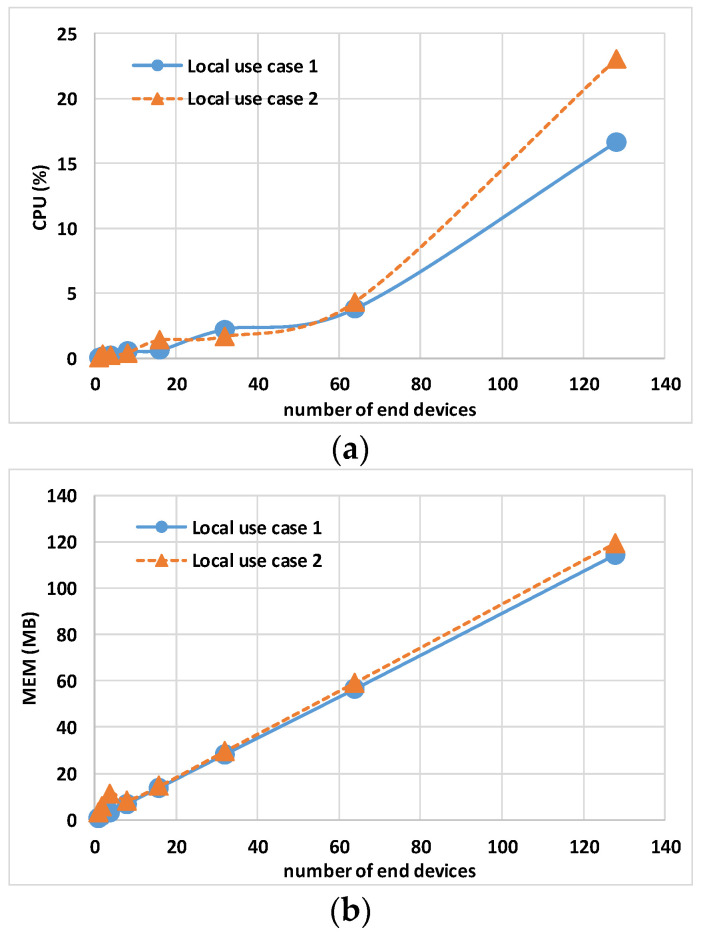

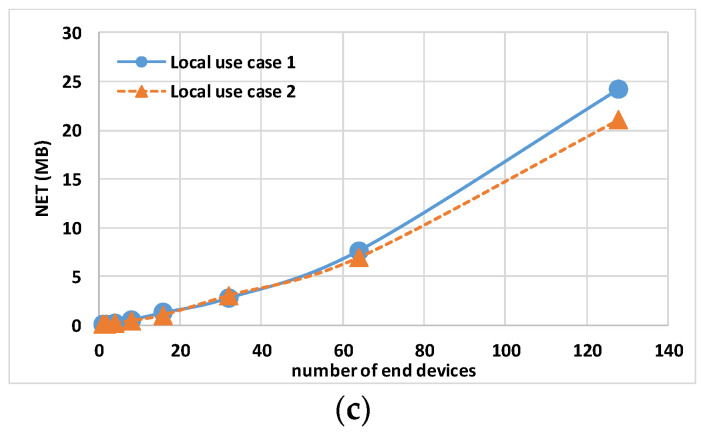
Local use case 1 and local use case 2 sum of end node containers’ 95th percentile of maximum host hardware utilization multiplied by the number of nodes N: (**a**) N × CPU, (**b**) N × MEM, and (**c**) N × NET.

**Figure 8:**
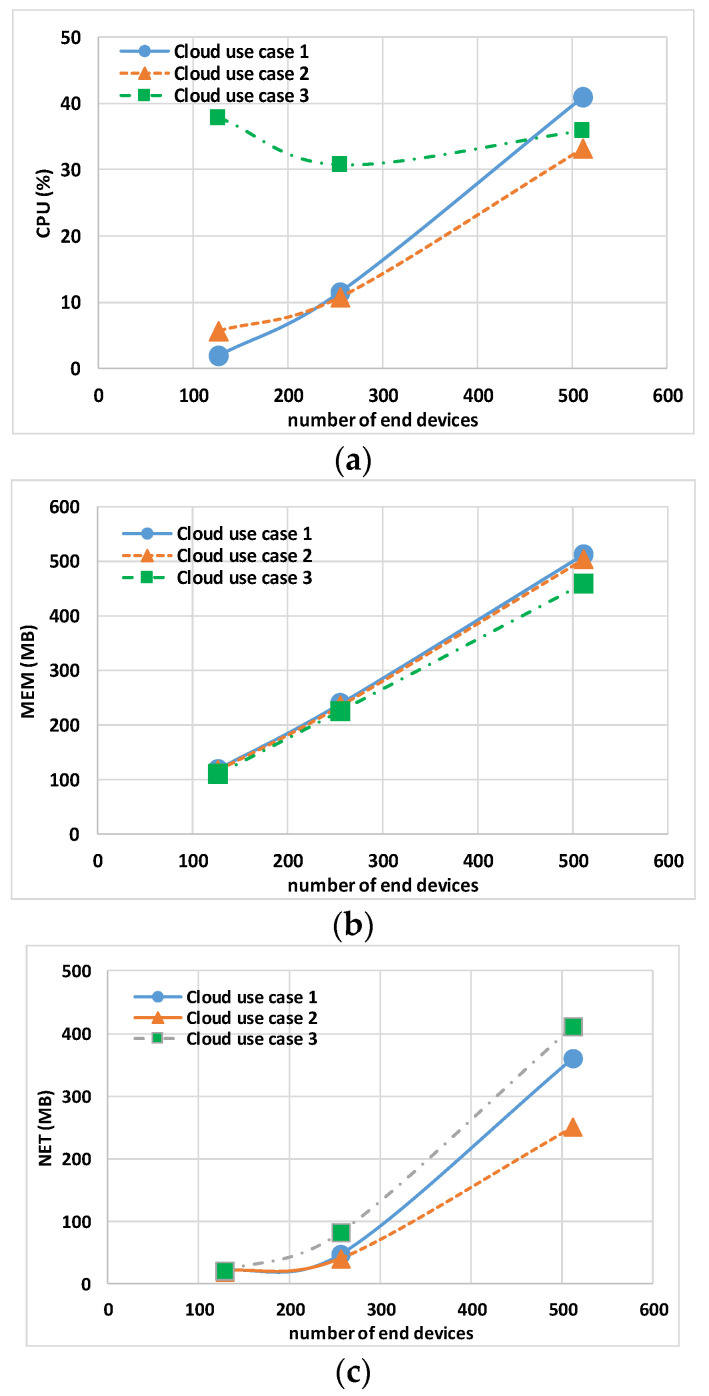
Cloud use case 1, cloud use case 2, and cloud use case 3 sum of end node containers’ 95th percentile of maximum host hardware utilization: (**a**) CPU, (**b**) MEM, and (**c**) NET.

**Figure 9:**
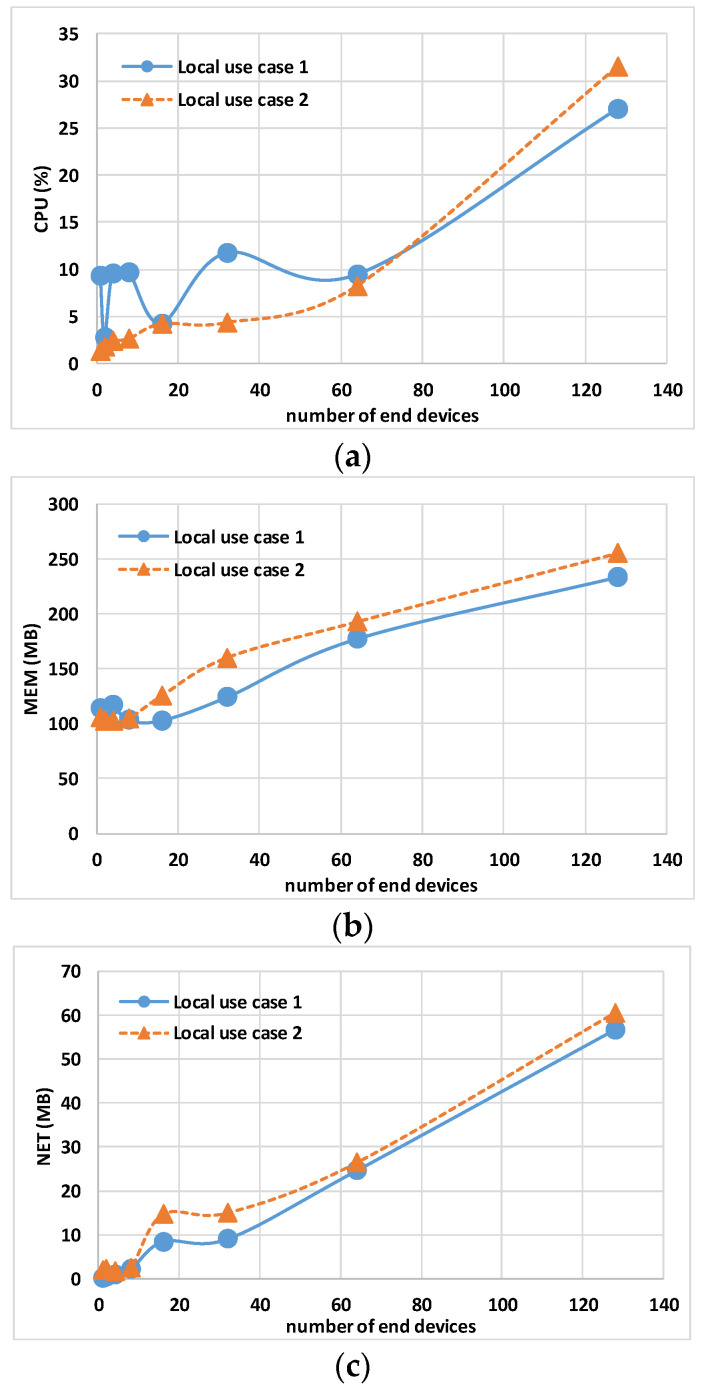
Local use case 1 and local use case 2 total containers’ host hardware utilization: (**a**) TCPU, (**b**) TMEM, and (**c**) TNET.

**Figure 10:**
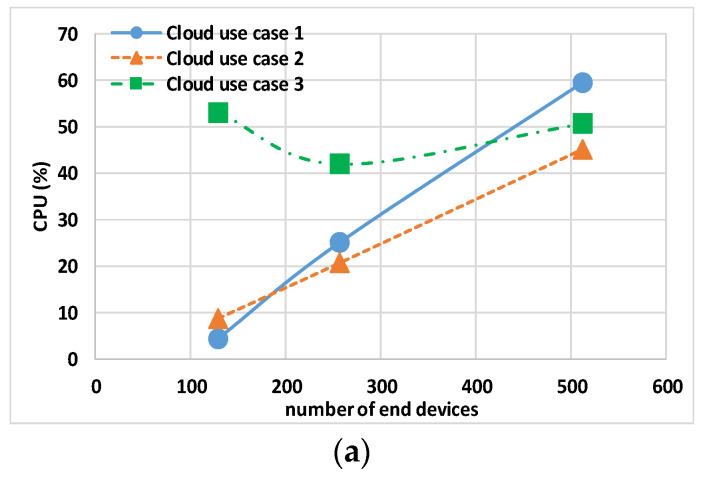

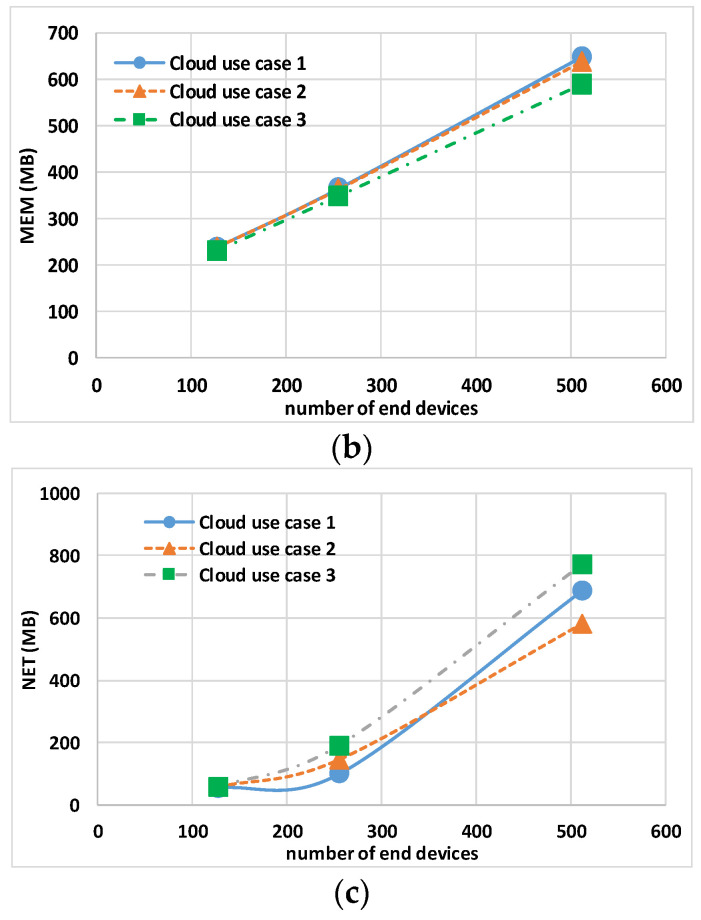
Cloud use case 1, cloud use case 2, and cloud use case 3 total containers’ host hardware utilization: (**a**) CPU, (**b**) MEM, and (**c**) NET.

The data in Table 4 and Table 5 show the maximum CPU, MEM, and NET values for the Controller, gateway, and Eclipse-mosquitto containers. Figure 7 and Figure 8 show the host hardware utilization for end nodes. The graphs are obtained by calculating the 95th percentile of the maximum value of all end nodes multiplied by the number of end nodes, N, involved in the experiment. Figure 9 and Figure 10 show the host hardware utilization for all the containers present in the VMy.

### 7.1. Emulation on Local Machines

Table 4 shows that for local use case 1, the Controller occupies the CPU for a longer duration. However, the trend is not monotonic as the number of nodes increases, likely due to the unpredictable behavior of the virtualization software (which is different from local use case 2). As a matter of fact, for local use case 2, the controller exhibits a growth based on the number of devices.

Similar trends are observed for memory usage and network traffic. Both the gateway and Eclipse-mosquitto exhibit similar trends across both use cases. The CPU usage of Eclipse-mosquitto is particularly noteworthy, as it experiences a sharp increase between 64 and 128 nodes. Specifically, for local use case 1, it rises from 1.56% to 5.95%, while for local use case 2, it increases from 0.5% to 5.69%. The most probable reason is the increase in topics and subscriptions that saturates the default settings of the mosquitto broker.

Figure 7 shows the host hardware utilization for all end nodes. While N × MEM usage and N × NET report a similar trend, the N × CPU usage shows a relevant difference when simulating 128 end nodes, respectively, 16.64% for local use case 1 and 23.04% for local use case 2. This difference is due to the fact that CPU usage has an inverse relationship with the host CPU performance: local use case 1 has a processor with a 2.30 GHz base clock, while local use case 2 has a 1.80 GHz processor.

Figure 9a shows the total host hardware utilization for all the containers. The TCPU usage for local use case 1 between 1 and 64 end nodes fluctuates; the reason is linked to the fluctuations of the Controller CPU usage described before (i.e., depending on the virtualization software). The TMEM usage shown in Figure 9b and the TNET usage shown in Figure 9c report a similar trend. Having a similar TMEM usage demonstrates that containers show the same computational load on the different hosts.

### 7.2. Emulation in Cloud

Table 5 shows the results for the Controller, gateway, and Eclipse-mosquitto in the cloud use cases. The first remark is related to cloud use case 3 that, different from others use cases, does not have a fixed processor; this use case exhibits a nonlinear CPU usage with the increase in the total number N of end nodes. For instance, the TCPU is more than 50% in the case of (just) N = 128. The second remark is about cloud use case 1 and cloud use case 2, where it is evident that when increasing the vCPUs of the cloud host machine, the maximum CPU usage of the single CPU decreases.

The analysis of end nodes’ indicators and the total usage indicators is shown in Figure 8a and Figure 10a, respectively. For less than 256 end nodes, cloud use case 1 shows a lower N × CPU of 1.92% and TCPU of 4.28%, while cloud use case 2 has an N × CPU of 5.76% and TCPU 8.76%. On the contrary, when increasing the end nodes to N = 512, lower values of N × CPU and TCPU are obtained with cloud use case 2. This leads to the conclusion that with Docker default settings, the better option seems to be to use the minimum number of CPUs that keeps the TCPU under 50%.

Regarding the MEM usage, Figure 8b and Figure 10b report N × MEM and the TMEM, respectively. The trend is similar for all the use cases, showing the independence of memory indicators from the type and number of vCPUs.

Regarding the NET usage, Figure 8c and Figure 10c show N × NET and the TNET, respectively. This indicator is dependent on the number of vCPUs (i.e., on the value of the TCPU); a low TNET for cloud use case 2 is visible especially with 512 end nodes, indicating that end nodes containers’ (due to a more powerful host) remain for less time waiting for closing MQTT transactions and thus they generate less traffic for “keep alive”.

**Figure 11:**
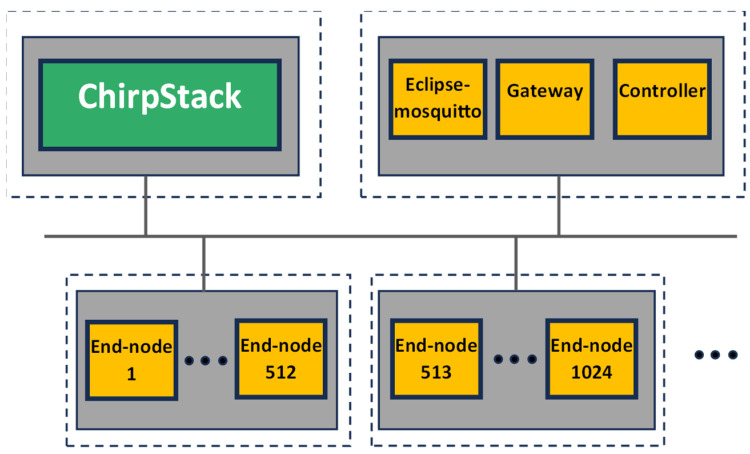
Modified architecture to accommodate more than 1024 containers.

As a final remark, it is worth noting that for cloud experiments only, N ∈ [128, 256, 512] end nodes are emulated on the same virtual machine. With the proposed architecture, Figure 6, the upper limit is 1024 containers on the same virtual machine. This limit is due to the maximum number of ports Linux will allow on a Linux bridge, which is 1K. This value is controlled by two hardcoded parameters in the Linux kernel (https://www.ibm.com/blog/docker-insane-scale-on-ibm-power-systems/, accessed on 4 March 2024). Figure 11 proposes a potential variation in the architecture to accommodate more than 1024 containers. The end nodes are distributed in different hosts, each with a virtual network. The Controller, end nodes, and gateway are in a separate virtual machine. Communication between the Controller, end nodes, and gateway is based on MQTT, requiring a new mapping of the Eclipse-mosquitto container port to the host port.

### 7.3. Emulation Costs for Cloud Use Cases

When dealing with cloud-based computing services, the cost of running the virtual machines is of the main concern. Table 6 displays the emulation costs for the cloud experiment. (Note: the reported costs refer to the current contract between Google Cloud and the authors of this paper, hence they are used only for reference purposes).

As first, it should be noticed that the experiment is divided into three distinct phases:

- Startup, which begins with the start of the containers by the docker command and ends when the last end node completes the join procedure with the backend;
- Emu, the phase of the experiment where data are exchanged with the backend and metrics are collected;
- Stop, the stop time of the experiment. The stop procedure is conducted by killing the container by the kill command.

The Startup and Stop phases are not useful for the emulation of the architecture but are needed to prepare the emulation. From the cost point of view, they can be considered as preparation costs.

From the results, it appears that Startup and Stop scale up with the increasing of nodes, but they do not decrease if the number of CPUs is increased. This means that the generally right strategy to reduce costs is to choose the cheapest virtual machine.

Last, it is worth noting that in use case 3, the costs are lower, but the reason is that the CPU type is variable from the time the emulation is conducted. Such a situation, as shown in the previous section, may pose some risks that the emulation fails due to CPU saturations.

In conclusion, the preparation costs never exceeded 18 min, and the emulation cost increases linearly with the emulation time of the experiments. Thus, it is easy to generalize the cost calculation of any experiment. For instance, the emulation of a full LoRaWAN architecture with 512 nodes costs as follows: for one hour, USD 0.14 for use case 1, USD 0.21 for use case 2, and USD 0.12 for use case 3; for one entire day, respectively, USD 2.7, USD 4.1, and USD 2.4.

### 7.4. Resuming Remarks

The computational weight of the emulation was evaluated locally with different hardware setups using a reduced number of nodes, ranging from 1 to 128. Upon analyzing the local scenarios, both use cases demonstrated comparable trends in memory and network usage. However, the CPU usage indicated a shorter utilization in local use case 1, which has a processor with a higher base frequency compared to local use case 2. As the number of nodes increases, it is important to monitor the CPU usage of the Eclipse-mosquitto container, which does not increase linearly. The comparison of the two use cases demonstrated that standard PCs can effectively emulate full-stack LoRaWAN systems with minimal computational load up to 128 nodes. This confirms that containerization introduces negligible overhead.

To assess scalability and achieve a number of nodes comparable to a smart city scenario, it was decided to evaluate the use of cloud platforms. Due to the limitations of the architecture in use, we tested up to 512 nodes. Use cases 1 and 2 differ only in the number of vCPUs. During the emulation, it was demonstrated that from the performance point of view, it is better to use a machine with more than two vCPUs only over 128 nodes, due to how Docker allocates containers by default. As the number of end nodes increases, the experiment showed that the maximum TCPU usage increases less in cloud use case 2 than in cloud use case 1. A more cost-effective solution was proposed with a machine that varies its CPU according to availability in cloud use case 3; unlike previous use cases, the experiment demonstrated that CPU usage is not dependent on the number of nodes. Additionally, comparing TMEM and TNET usage across different scenarios provides insights into resource utilization patterns within cloud environments, highlighting the efficiency of container management in cloud use case 2.

From the cost point of view, the emulation approach allows for an affordable way to support the full development of the application layer and to test complex software as it will run in the final deployment. The cost of the emulation of the considered use case scenario with 512 nodes is between USD 0.12 and USD 0.21 per hour including preparation costs. It is also possible to stress the impact of setting up the emulated scenario, in terms of preparation costs that can be relevant if the duration of the emulation is short.

## 8. Conclusions

In this study, the focus was on characterizing simulation systems capable of emulating innovative full-stack end-to-end LoRaWAN architectures, given the recent trend towards a fully disaggregated and containerized approach. Furthermore, the analysis traversed the landscape of both on-premises and cloud-based environments, providing a comparative perspective for analyzing resource usage and performance dynamics.

The paper started by highlighting the differences between traditional network simulations and the network emulation. Successively, the work characterized simulation systems that can emulate an entire end-to-end LoRaWAN architecture built with the innovative end-to-end full-stack containerization. In this part of the study, fundamental evaluation metrics for comparing different implementations were provided, and then they were applied to five use cases inspired by a smart city scenario.

In conclusion, the results of this study demonstrate the effectiveness of containerization in emulating full-stack LoRaWAN architectures across both local and cloud-based environments. The findings emphasize the scalability, efficiency, and cost of container management, providing valuable insights into optimizing resource utilization and performance in a smart cities scenario before deploying real hardware devices.

## Figures and Tables

**Table 1:** Local host and virtual machine configuration.

	Local Use Case 1	Local Use Case 2
Host Processor	AMD Ryzen 5 5600U	Intel Core(TM) i7-8550U
Host Graphics	Radeon Graphics 2.30 GHz	Off-board Radeon Graphics 530
Host Memory	40.0 GB	16.0 GB
Host OS	Windows 11 Pro Version 22H2 (OS build 22621.2715)	Windows 11 Home, Version 22H2 (OS Build 22621.2715)
Virtualization SW	VMware Workstation 17.0.2 Pro	VirtualBox 7.0.6
VMx Processor	One-Core	Dual-Core
VMx Memory	8.0 GB	2.0 GB
VMx OS	Ubuntu 20.04.6 LTS	Debian 12 stable
VMx Kernel Version	5.4	6.1.55-1
VMx Network Interface	Two (LAN and Host Only)	Two (LAN and Host Only)
VMxApplication	Docker engine version 19.03.15 and Docker-Compose version 1.28.5	Docker engine version 24.0.5 and Docker-Compose version 1.29.2
VMy Processor	One-Core	Dual-Core
VMy Memory	8.0 GB	4.0 GB
VMy OS	Ubuntu 20.04.6 LTS	Ubuntu 22.04 LTS
VMy Kernel Version	5.4	6.2.0-36-generic
VMy Network Interface	Two (LAN and Host Only)	Two (LAN and Host Only)
VMy Application	Docker engine version 19.03.15 and Docker-Compose version 1.28.5	Docker engine version 24.0.5 and Docker-Compose version 1.29.2

**Table 2:** Cloud virtual machine configuration.

	Cloud Use Case 1	Cloud Use Case 2	Cloud Use Case 3
VMx Series	N2	N2	E2
VMx Processor	Intel Cascade Lake, One-Core, 2 vCPUs	Intel Cascade Lake, One-Core, 2 vCPUs	Depending on availability, One-Core, 2 vCPUs
VMx Memory	8.0 GB	8.0 GB	8.0 GB
VMx OS	Debian 12 bookworm	Debian 12 bookworm	Debian 11 bullseye
VMx Kernel Version	v20240110	v20240110	v20240110
VMx Application	Docker engine version 25.0.2 and Docker compose version 2.24.5	Docker engine version 25.0.2 and Docker compose version 2.24.5	Docker engine version 25.0.2 and Docker compose version 2.24.5
VMx Price	0.11 USD/h	0.11 USD/h	0.10 USD/h
VMy Series	N2	N2	E2
VMy Processor	Intel Cascade Lake, One-Core, 2 vCPUs	Intel Cascade Lake, Two-Core, 4 vCPUs	Depending on availability, One-Core, 2 vCPUs
VMy Memory	8.0 GB	8.0 GB	8.0 GB
VMy OS	Debian 12 bookworm	Debian 12 bookworm	Debian 11 bullseye
VMy Kernel Version	v20240110	v20240110	v20240110
VMy Application	Docker engine version 25.0.2 and Docker compose version 2.24.5	Docker engine version 25.0.2 and Docker compose version 2.24.5	Docker engine version 25.0.2 and Docker compose version 2.24.5
VMy Price	0.11 USD/h	0.17 USD/h	0.10 USD/h

**Table 3:** Suitability of LoRaWAN emulation solution for different requirements and use cases.

	Local Use Case 1	Local Use Case 2	Cloud Use Case 1	Cloud Use Case 2	Cloud Use Case 3
Realistic functionality	+++	+++	+++	+++	+++
Realistic timing	--	--	+	+	-
Realistic traffic	++	++	++	++	++
Topology flexibility	+++	+++	+++	+++	+++
Reproducibility	+++	+++	+++	+++	+++
Cost	--	--	++	++	+++
Isolation level	+	+	+	+	+
Different OSs	--	--	--	--	--
Setup time	-	-	-	-	-
Channel modeling	not available	not available	not available	not available	not available

**Table 4:** Local use case 1 and local use case 2 Controller, gateway, and Eclipse-mosquitto containers’ maximum host hardware utilization.

	N	Local Use Case 1			Local Use Case 2		
		Controller	Gateway	Eclipse-Mosquitto	Controller	Gateway	Eclipse-Mosquitto
CPU (%)	1	7.89	1.18	0.21	0.2	0.94	0.13
	2	0.98	1.46	0.21	0.32	1.02	0.145
	4	7.68	1.13	0.5	1.36	0.685	0.135
	8	6.85	1.38	0.86	0.9	1.025	0.27
	16	2.02	1.25	0.4	1.11	1.14	0.57
	32	7.2	1.62	0.69	1.01	1.25	0.415
	64	2.98	1.07	1.56	1.79	1.575	0.5
	128	2.85	1.57	5.95	1.405	1.415	5.69
MEM (MB)	1	110.9	0.941406	0.66015625	99.05	3.242	0.71875
	2	109.3	0.957031	0.6640625	93.39	3.27	0.7265625
	4	111.2	1.027	0.6640625	89.59	1.066	0.7265625
	8	94.07	1.102	0.6953125	94.96	1.352	3.094
	16	86.53	1.238	0.7265625	109.3	1.195	0.75390625
	32	93.3	1.453	0.76171875	124.4	5.715	4.527
	64	118	0.855469	1.883	131.3	1.926	0.8828125
	128	115.4	2.957	1.016	132.9	2.25	1.078
NET (kB)	1	57	78.5	121	1855.48	61.5	88.3
	2	65.6	137.9	229.9	1764.28	107	174.5
	4	76.7	240.6	441	1003	182.1	332
	8	402.4	443	864	1113	320	666
	16	4410.44	905	1915.56	11,560.96	694	1497
	32	584	1676.96	4044.8	5629.44	1800.64	4638.72
	64	3860.04	9830.4	3317.76	7598.08	2908.16	9113.6
	128	1149	7137.28	24,135.68	10,455.04	4761.6	24,371.2

**Table 5:** Cloud use case 1 and cloud use case 2 Controller, gateway, and Eclipse-mosquitto containers’ maximum host hardware utilization.

	N	Cloud Use Case 1			Cloud Use Case 2			Cloud Use Case 3		
		Controller	Gateway	Eclipse-Mosquitto	Controller	Gateway	Eclipse-Mosquitto	Controller	Gateway	Eclipse-Mosquitto
CPU (%)	128	1.45	0.515	0.395	0.6475	0.195	2.1575	2.205	0.97	12.185
	256	1.51	0.51	11.425	0.5775	0.345	8.8575	2.255	0.9	8.08
	512	1.125	0.515	16.84	0.54	0.2375	10.9425	1.63	0.95	12.2
MEM (MB)	128	116	2.883	1.066	115.5	2.844	1.324	117.5	2.746	1.016
	256	122.3	2.902	1.426	119.9	6.164	1.684	116.5	4.582	2.367
	512	127	7.93	2.125	121.4	8.355	4.93	115.8	8.227	4.48
NET (kB)	128	2575.8	6676.48	23,951.36	2533.08	6574.08	26,931.2	2540.08	6502.4	26,419.2
	256	5712.32	6973.44	39,833.6	10,547.2	18,216.96	75,161.6	5109.76	13,066.24	90,726.4
	512	10,291.2	24,401.92	291,840	14,090.24	26,091.52	288,768	10,229.76	24,954.88	325,632

**Table 6:** Cloud use case 1 and cloud use case 2 Controller, gateway, and Eclipse-mosquitto containers’ maximum host hardware utilization.

N	Cloud Use Case 1 (0.11 USD/h)				Cloud Use Case 2 (0.17 USD/h)				Cloud Use Case 3 (0.10 USD/h)			
	Startup	Emu	Stop	Cost	Startup	Emu	Stop	Cost	Startup	Emu	Stop	Cost
128	38 s	15 m	19 s	USD 0.03	38 s	15 m	19 s	USD 0.04	54 s	15 m	20 s	USD 0.03
256	3 m 14 s	15 m	47 s	USD 0.05	3 m 15 s	15 m	41 s	USD 0.06	2 m 2 s	15 m	49 s	USD 0.03
512	15 m 21 s	15 m	2 m 25 s	USD 0.06	13 m 1 s	15 m	2 m 7 s	USD 0.09	8 m 47 s	15 m	2 m 6 s	USD 0.04

## Data Availability

The code is available at https://github.com/Gaffurini97/Sensor_LoRaWAN_emulation (accessed on 21 March 2024). Licenses may apply to some parts of the code.
